# Elevated OCT1 participates in colon tumorigenesis and independently predicts poor prognoses of colorectal cancer patients

**DOI:** 10.1007/s13277-015-4080-0

**Published:** 2015-10-04

**Authors:** Yu-peng Wang, Guo-he Song, Jian Chen, Chao Xiao, Chao Li, Lin Zhong, Xing Sun, Zhao-wen Wang, Gui-long Deng, Fu-dong Yu, Ying-ming Xue, Hua-mei Tang, Zhi-hai Peng, Xiao-liang Wang

**Affiliations:** 1 Department of General Surgery, Shanghai General Hospital, School of Medicine, Shanghai Jiao Tong University, Shanghai, China; 2 Department of Pathology, Shanghai General Hospital, School of Medicine, Shanghai Jiao Tong University, Shanghai, China

**Keywords:** Colorectal cancer, OCT1, Prognosis, Proliferation

## Abstract

Octamer transcription factor 1 (OCT1) was found to influence the genesis and progression of numerous cancers except for colorectal cancer (CRC). This study tried to explore the role of OCT1 in CRC and clarify the association between its expression and patients’ clinical outcome. Transcriptional and post-transcriptional expression of OCT1 was detected in CRC cancerous tissues and paired normal mucosae by real-time PCR as well as immunohistochemistry. Moreover, the effect of OCT1 knockdown on CRC cell proliferation was investigated both in vitro and in vivo using Cell Counting Kit-8 assay, colony-forming assay, and mouse tumorigenicity assay. Expression of OCT1 was found to be elevated in CRC. Suppression of OCT1 significantly inhibited CRC cell proliferation both in vitro and in vivo. Furthermore, upregulated level of OCT1 was significantly associated with N stage, M stage, and American Joint Committee on Cancer (AJCC) stage (*P =* 0.027, 0.014, and 0.002, respectively) as well as differential degree (*P =* 0.022). By using multivariate Cox hazard model, OCT1 was also shown to be a factor independently predicting overall survival (OS; *P* = 0.013, hazard ratio = 2.747, 95 % confidence interval 1.125 to 3.715) and disease-free survival (DFS; *P* = 0.004, hazard ratio = 2.756, 95 % confidence interval 1.191 to 4.589) for CRC patients. Our data indicate that OCT1 carries weight in colorectal carcinogenesis and functions as a novel prognostic indicator and a promising target of anti-cancer therapy for CRC.

## Introduction

Colorectal cancer (CRC) is among the most prevalent cancers in the world with annual new cases exceeding 100,000 [1]. In spite of improvements of surgical and adjuvant treatment approaches, the long-term survival associated with its malignancy is not satisfactory because of tumor recurrence and metastasis [2, 3]. Thus, it is clinically valuable to identify genes that function in germination and progression of CRC and their efficacy for predicting prognosis.

Octamer transcription factor 1 (Oct1; gene symbol *pou2f1*), a homeologous transcription factor of Oct4 [4], is comprised in the Pit-Oct-Unc (Pit1, Oct1/2, Unc86) family, which possesses similar in vitro DNA binding specificity [5]. Through binding to the canonical DNA motif (ATGCAAAT), OCT1 affects the transcription of a number of genes, like the immunoglobulin genes in B lymphocytes [6, 7], some interleukins [8–10], and Pit-1 [11]. As a *cis*-factor, OCT1 is also required in embryogenesis and maintaining stem cell function in vivo [12]. Maddox J et al. [13] had highlighted new target genes regulated by OCT1, such as *Abcg2* and *Abcb1*, which are in accordance with its effects in stem cells. Besides, it has been demonstrated that OCT1 was discovered to be over-expressed in gastric cancer (GC) [14, 15], cervical cancer [16], and prostate cancer [17, 18]. Additionally, by recognizing the CDX2 promoter, OCT1 is thought to boost malignancy of both pancreatic and intestine cell lines [19], and studies on p53-deficient mice revealed that dysfunction of OCT1 restrains in vitro tumor transformation and in vivo tumorigenicity via metabolic procedure shift [20]. Nevertheless, in GC cells, OCT1 was uncovered to lose the ability to activate CDX2 transcription despite that it could be recruited to the CDX2 promoter [15, 21]. In spite of recent studies exerting to elucidating OCT1 as a *cis*-factor in malignancies, the role of OCT1 in colorectal carcinogenesis remains in its infancy and efforts should be made to clarify the associations between OCT1 level and clinicopathological features of CRC.

These observations prompted us to investigate the expression of OCT1 in human colorectal cancer by utilizing real-time PCR and immunohistochemistry (IHC), to figure out the relationship between the OCT1 expression and clinical parameters and explore its potential role in malignant colonic hyperplasia both in vitro and in vivo.

## Material and methods

### Patients and clinical samples

The present research was sanctioned by the Institutional Research Ethics Committee of Shanghai General Hospital, and informed consent was signed by all the 98 patients, during 2007–2009, diagnosed with CRCs affirmed by clinical resection and pathology. No patients accepted either chemotherapy or radiotherapy before the operation. The sixth American Joint Committee on Cancer (AJCC) staging method was also applied for tumor staging by two pathologists. Cancer tissues and adjacent normal mucosae (at least 10 cm from the primary tumor) surgically removed from colon cancer patients were immediately frozen in liquid nitrogen and stored at −80 °C. Formalin-fixed matched noncancerous tissue, primary tumor tissue, and metastatic lymph nodes were embedded in paraffin for later immunohistochemistry (IHC) studies after a series of dehydration.

### RNA isolation and real-time PCR (qPCR)

Total RNA was extracted from cancerous tissues and paired normal mucosae from 38 colon cancer patients according to protocols of the manufacturer (TRIzol, Invitrogen, USA). Complementary DNA was synthesized using 1 μg RNA by RevertAid™First Strand cDNA Synthesis Kit (Fermentas, USA). Quantitative PCR reaction was done on a ViiA™ 7 Real-Time PCR System (Life Technology, USA) with SYBR® Premix DimerEraser™ (Perfect Real Time) (Takara, Japan) in accordance with the manufacturer’s instructions. Each experiment was performed in triplicate. Primer sequences were as follows: *pou2f1* forward 5′-AAAAGAAATCAACCCACCAAGC-3′ and reverse 5′-TGTGGTTCGGAACACTGATCG-3′, *Gapdh* forward 5′-GGCCAAGGTCATCCATGACAA-3′ and reverse 5′-TCTTCTGACACCTACCGGGGA-3′, *Ki67* forward 5′-TTCGCAAGCGCATAACCCA-3′ and reverse 5′-AACCGTGTCACAGTGCCAAA-3′, and *cyclin D1* forward 5′-GCTGCGAAGTGGAAACCATC-3′ and reverse 5′-CCTCCTTCTGCACACATTTGAA-3′. The cycling conditions were as follows: 95 °C, 2 min, 1 cycle, and 95 °C, 10s, and 60 °C for 30 s followed by 30 s at 72 °C, 40 cycles. Amplification product’s specificity was confirmed by the exhibition of a singlet in the melting curve and electrophoresis.

### IHC

After dewaxing and rehydrating in a graded series of ethanol, sections (4 μm thick) were blocked with 3 % H_2_O_2_. Then, slides were prepared for antigen retrieval process with 0.01 M sodium citrate solution (pH 6.0). Primary anti-human rabbit polyclonal antibody against OCT1 (1:200; Abcam, USA) was used for IHC staining, followed by incubation with the horseradish peroxidase (HRP)-conjugated secondary detection antibody (Dako Cytomation, Glostrup, Denmark). Negative control was treated with PBS in the place of anti-OCT1 antibody. Blinded evaluation of immunoreactivity was executed independently by two pathologists. The measurement was calculated by both the intensity and the area of staining. Density of tintage was ranked as follows: 0, no staining; 1, mild staining; 2, moderate staining; and 3, intense staining. Area of staining was sorted as follows: 0, no positive cells; 1, <10 % of extent positive; 2, 10–50 % of extent positive; and 3, >50 % of extent positive. The final staining score was obtained by multiplying the intensity score by the extent score. The samples were classified into two groups by the final scores: low (0–4) and high (5–9).

### Cell culture and plasmids

Human colorectal cancer cells HCT116 and RKO were purchased from American Type Culture Collection (Rockville, MD). Cultured at 37 °C under a moist air with 5 % CO_2_, all cell lines were maintained with Dulbecco’s modified Eagle medium with 10 % FBS (Gibco, USA). The short hairpin RNA (shRNA) plasmid for OCT1 and the control-shRNA plasmid were purchased from Obio Technology (Shanghai, China). For plasmid transfection, 4 × 10^4^ cells per well in six-well plates were cultured overnight and then transfected with plasmids utilizing Lipofectamine 2000 (Invitrogen, CA). Stable clones of HCT116 and RKO cells expressing OCT1 shRNA or control-shRNA were obtained by puromycin selection. The shRNA target sequence was 5′-GCCTTGAACCTCAGCTTTAAG-3′ and 5′-GGAATTAATTGCATGAATTAG-3′ (Oct1-shRNA and control-shRNA, respectively).

### Western blot

Total protein was isolated using RIPA solution (Beyotime Biotechnology, Jiangsu, China) according to the instructions. BCA protein assay kit (Beyotime Biotechnology, Jiangsu, China) was applied to measure the concentration of the protein. The same quantity of protein (30 μg) was spotted into 10 % SDS-polyacrylamide gel for electrophoresis and then electrotransferred onto PVDF membranes. Membranes were immersed at room temperature for 1 h in 5 % fat-free milk solution with 0.1 % Tween 20, and then they were hatched with antibodies against Oct1(1:500, Abcam) or Ki67 (1:100, Abcam) or cyclin D1(1:100, Abcam) overnight, and then incubation with HRP-tagged detection antibody (1:5000, Abgent) was performed. Signal visualization was performed using ECL reagent (Pierce Biotechnology, USA), and grayscale analysis was carried out by Quality One (Bio-Rad, USA).

### Proliferation assay

Cell Counting Kit-8 kit (Dojino, Japan) was used to evaluate cell proliferation according to the manufacturer’s protocols. In brief, 96-well plates were seeded with 2 × 10^3^ cells per well in triplicate. At the appropriate time (24, 48, 72, 96 h), each well was incubated for 1.5 h with 10 μl CCK8 solution at 37 °C with moist air with 5 % CO_2_. Then, absorbance at 450 nm was detected on a Gen5 microplate reader (BioTek, USA).

### Colony formation assays

For plate colony formation assays, six-well plates were seeded using 1000 log-phase cells per well and cultured at 37 °C with 5 % CO_2_ concentration atmosphere for 2 weeks. After fixed by methanol for 15 min, the cells were then stained with Giemsa solution for 20 min followed by colony photographing and counting. All assays were independently performed in triplicate.

### Mice xenograft implantation

Stably transfected sh-OCT1 or sh-control vector of HCT116 or RKO cells were injected subcutaneously in the flank of 6-week-old nude mice (1 × 10^6^ cells/mouse, five mice/group) as previously described [22]. All the procedures involving mice are in accordance with the Shanghai Jiaotong University Affiliated Shanghai General Hospital Animal Care guidelines. Endeavors were made to the greatest extent to make animals suffer minimally, or to abate the amount of animals to the best exploiture. Tumor weight and bulk were measured every 7 days, and mice were killed at 42 days after implantation.

### Statistical analysis

Data from two experimental groups were analyzed by using two-tailed Student’s *t* test. Pearson χ^2^ test or Fisher’s exact test was used to compare categorical data. Kaplan–Meier method was applied to calculate cumulative survival ratio. Hazard proportion of univariate and multivariate hazard for the clinical factors of CRC patients was computed by establishing a Cox hazard model. All data analyses were performed by SPSS software 19.0 (SPSS, Chicago, IL, USA). A *P* value <0.05 was considered to be statistically significant.

## Results

### OCT1 expression levels are significantly upregulated in human colorectal cancer

**Fig. 1:**
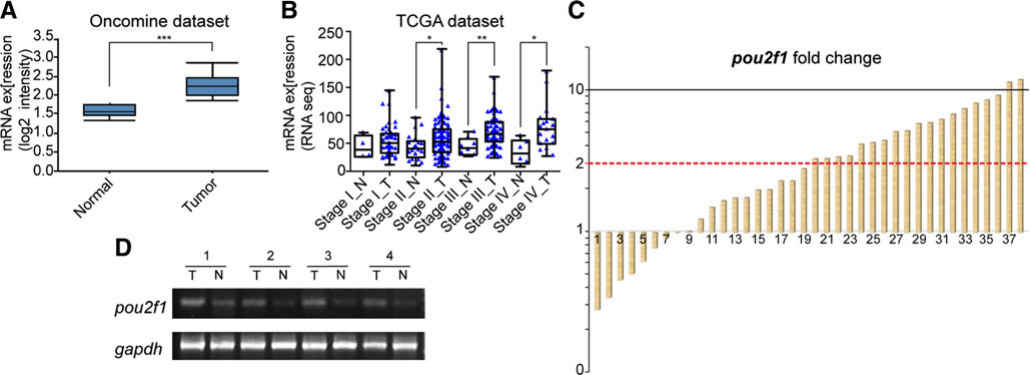
Levels of OCT1 in tumorous colon mucosae and matched normal tissues. **a** Exhibition of OCT1 expression of colorectal carcinoma and normal specimens from Hong colorectal statistics in Oncomine dataset (*t* test, ***P < 0.001); **b** statistical analysis of OCT1 mRNA levels in normal tissue and CRC mucosa according to AJCC stage from TCGA dataset (Mann–Whitney test, *P < 0.05, **P < 0.01); **c** relative expression of OCT1 (gene symbol *pou2f1*) in 38 paired tumorous samples compared with normal samples. Fold change was calculated by 2^−ΔΔCT^ method; **d** RT-qPCR product electrophoreses of OCT1 amplification in four paired CRC tissues

We first browse Oncomine (www.oncomine.org) to acquire OCT1 expression in colorectal carcinoma and found that its level was significantly high in tumor tissues compared with the related normal mucosae (Fig. 1a). Further, we analyzed data from The Cancer Genome Atlas (TCGA) according to AJCC stage. The OCT1 level was augmented in stages II, III, and IV but not in stage I (Fig. 1b). Then, 38 paired specimens were analyzed for OCT1 messenger RNA (mRNA) level and protein level; 19 (50 %) colorectal cancers revealed a more than twofold accumulation in OCT1 mRNA expression compared with related normal tissues (Fig. 1c, d). These data indicated that OCT1 expression is commonly elevated in human CRC.

### OCT1 augment is associated with poor survival of CRC patients

**Fig. 2:**
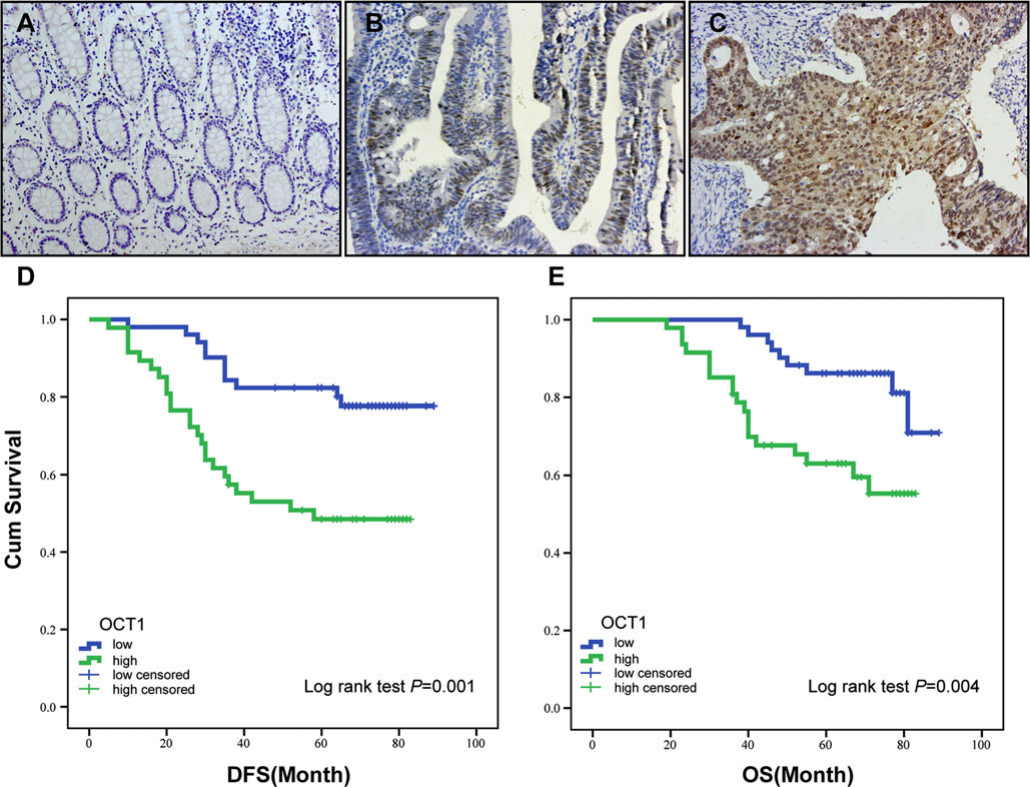
Immunohistochemical staining for OCT1 expression and Kaplan–Meier plots for survival by log-rank test. **a** Negative staining of OCT1 in normal mucosa, ×200; **b** positive for OCT1 expression in moderately differentiated cancer, ×200; **c** OCT1-positive of the poorly differentiated tumor, ×200; **d**, **e** patients’ disease-free survival (DFS) and overall survival (OS) according to OCT1 rank measured by immunohistochemistry; cum survival was for cumulative survival

We further evaluated the relationship between OCT1 level and numbers of clinicopathological parameters of CRC patients. Among the 98 samples, 66 (67.35 %) showed negative staining in paired normal mucosae (Fig. 2a). In contrast, upregulated OCT1 expression was apparent in colorectal tumors, with low staining in 40 (40.82 %) specimens and high staining in 58 (59.18 %) specimens. Summarization of associations between OCT1 and clinical parameters is shown in Table 1. Positive tintage was principally watched in the nucleus of tumor cells in samples (Fig. 2b, c). Upregulated OCT1 level was significantly associated with N stage (*P* = 0.027), M stage (*P =* 0.014), AJCC stage (*P =* 0.002), and differential grade (*P* = 0.022, Table 1). However, no association was found between OCT1 and sex, age, location, or vascular invasion. Notably, augmented expression of OCT1 is strongly associated with poor survival of patients with CRC. The Kaplan–Meier analysis revealed that patients with high levels of OCT1 possessed shorter overall survival (OS) time and disease-free survival (DFS) time than subjects with low levels of OCT1 (*P* = 0.004 and *P* = 0.001, respectively, Fig. 2d, e). Survival analysis using multivariate Cox model adjusted for AJCC stage, tumor location, TNM stage, vascular invasion, and sex and age of characters suggested strong association between OCT1 level and shorter OS (*P* = 0.013, hazard ratio (HR) = 2.747, 95 % confidence interval (CI) 1.125 to 3.715) and DFS (*P* = 0.004, HR = 2.756, 95 % CI 1.191 to 4.589) as well (Table 2). These findings consistently imply OCT1 as a potential prognostic indicator for CRC.

**Table 1:** Correlation between OCT1 expression and clinicopathological characteristics

	OCT1 protein expression		*P* value
	Low (*N* = 40)	High (*N* = 58)	
Age			0.076
<65	21	20	
≥65	19	38	
Gender			0.867
Male	20	30	
Female	20	28	
Location			0.737
Right	20	27	
Others	20	31	
T stage			0.700
T1 + T2	25	34	
T3 + T4	15	24	
N stage			0.027*
N0	27	26	
N1 + N2	13	32	
M stage			0.014*
M0	40	50	
M1	0	8	
AJCC stage			0.002*
I + II	29	24	
III + IV	11	34	
Differentiation			0.022*
Moderate + well	28	27	
Poor	12	31	
Vascular invasion			0.418
No	24	30	
Yes	16	28	

*P* value derived from chi-square test or Fisher’s test

*AJCC* American Joint Committee on Cancer

*P < .005

**Table 2:** Multivariate analysis of metastasis-free survival (DFS) and overall survival (OS) of 98 colon cancer patients

Variable	Mutivariate analysis (DFS)			Mutivariate analysis (OS)		
	*P* value	HR	CI (95 %)	*P* value	HR	CI (95 %)
AJCC stage (I/II vs III/IV)	0.048*	1.911	1.005–3.634	0.019*	2.622	1.171–5.870
Differentiation (low vs high)	0.010*	2.004	1.184–3.392	0.017*	2.196	1.151–4.190
N (N1 + N2 vs N0)	0.026*	2.359	1.107–5.027	0.001*	4.311	1.776–10.461
M (M1 vs M0)	0.016*	12.792	1.617–20.202	0.006*	3.363	2.706–4.138
OCT1 (low vs high)	0.004*	2.756	1.191–4.589	0.013*	2.747	1.125–3.715

*AJCC* American Joint Committee on Cancer, *HR* hazard ratio, *CI* confidence interval

*P < .005

### Inhibition of colorectal cancer cell growth by shRNA induced downregulation of OCT1 expression both in vitro and in vivo

**Fig. 3:**
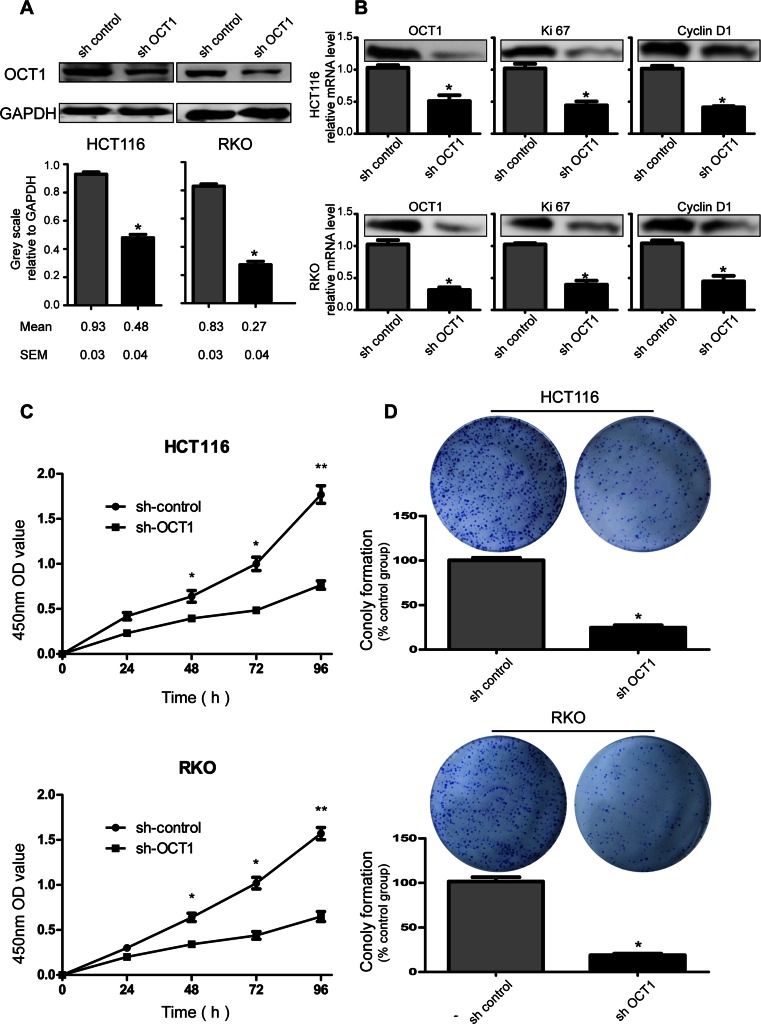
OCT1 knockdown inhibits cancer cell proliferation. **a** Western blot analyses of OCT1 level in stable knockdown HCT116 and RKO cell lines. Grayscale values were evaluated (*n* = 3, *P < 0.05); **b** expression of proliferation-related genes was inhibited in OCT1 knockdown cells according to real-time PCR and western blot (*n* = 3; * *P* < 0.05); **c**, **d** Effects of OCT1 knockdown on cell growth were evaluated by Cell Counting Kit-8 assays (**c**) and plate colony formation assays (**d**) (*n* = 3; * *P* < 0.05, **P < 0.01)

**Fig. 4:**
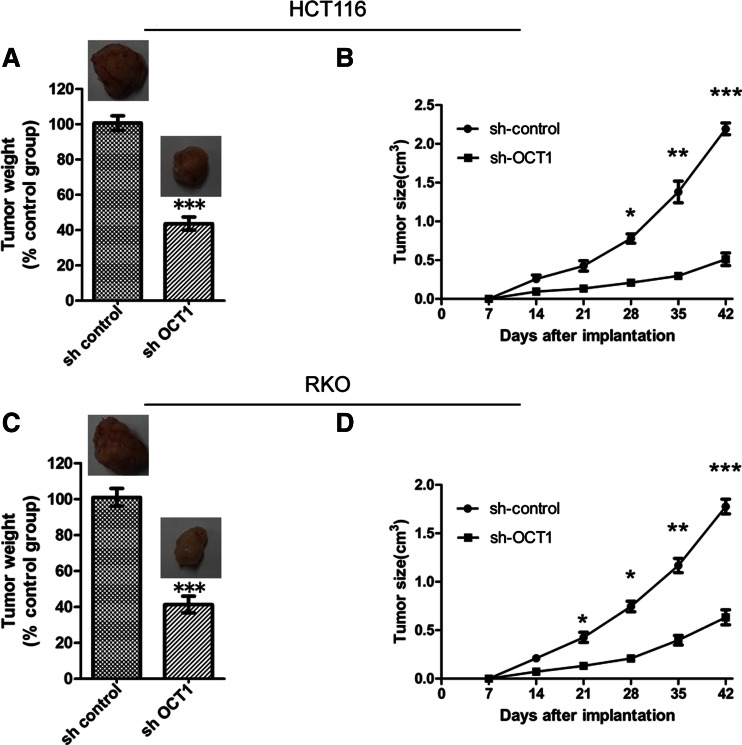
Tumor weight and volume of mice treated with sh-control or sh-OCT1 colon cancer cells. **a**, **c** Weights of tumor in mice injected with sh-control or sh-OCT1 transfected HCT116 and RKO cells, respectively; **b**, **d** tumor sizes in mice injected with sh-control or sh-OCT1 transfected HCT116 and RKO cells, respectively (*n* = 5; tumor weight of control group was arbitrarily set to be 1, *P < 0.05, **P < 0.01, ***P < 0.001)

To determine the role of OCT1 in colorectal cancer cell propagation, we treated HCT116 and RKO cells with OCT1 shRNA. We confirmed the efficacy of knockdown of OCT1 expression by using qPCR and Western blot (Fig. 3a). To determine the effects of OCT1 knockdown on CRC cell propagation, the expression of proliferation-related genes (*Ki67*; *cyclin D1*) was detected by quantitative PCR (Fig. 3b). The expression of both Ki67 and cyclinD1 mRNA was downregulated in OCT1-shRNA cells. Next, we determined cell viability by using CCK-8 assay and colony formation ability (Fig. 3c, d). As shown in Fig. 3c, OCT1 knockdown was associated with significantly decreased cell reproduction compared with cells transfected with control-shRNA. Furthermore, OCT1 knockdown in CRC cells consistently reduced the colony formation ability compared with mock-shRNA cells (*P* < 0.01). These in vitro data suggested that OCT1 contributes to being critical in the propagation of colorectal cancer cells. Thus, we determined to explore the capacity of multiplication of knockdown OCT1 colon cancer cells in vivo. Compared with those injected with sh-control cells, mice injected with sh-OCT1 cells exhibited tardive tumor morbidity and reduced tumor growth (Fig. 4).

## Discussion

OCT1, located in 1q24.2, is one of the first discovered transcription factors (TFs) of POU orthologous family, and it not only regulates levels of housekeeping genes as histone *H2B* and tissue-specific gene as *B29* [23] but also harbors the capacity of pro-proliferative function in tumors [24]. Although the OCT1 transcription factor was found to be over-expressed in many cancer cells [21, 25] and there exist considerable investigations of its role in tumors [14–18], in CRCs, the bio-functional and prognostic value of OCT1 has rarely been expounded.

We uncovered aberrantly upregulated transcriptional expression of OCT1 in CRC using our own tissue bank as well as two public databases (Oncomine database and TCGA database) and confirmed its post-transcriptional level in the established bank. Analysis of OCT1 level and patients’ clinical outcomes suggested that those with high OCT1 expression had poorer survival than those with low OCT1 expression. Additionally, in accordance with discoveries of Perri et al. in thyroid cancer cells [26], OCT1-interfering CRC cell lines exhibited impaired proliferative potency both in vitro and in vivo. These observations direct us to believe that OCT1 carries weight in colonic carcinogenesis and malignant progression and it is of great value as a predictor for prognoses of colorectal cancer patients.

To the best of our knowledge, it is the first time that OCT1 transcription factor is implied as a promising predictor of the prognoses of patients with colon cancer, and interfering OCT1 expression restrains hyperplasia in CRC oncogenesis. Interestingly, Fang et al. found that, in gastric cancer, elevated OCT1 level facilitated canonical extracellular-signal-regulated kinase (ERK) signaling pathway by transactivating synbindin which binds to ERK DEF domain, resulting in activation of ERK substrates ELK1 and RSK which finally leads to increased proliferative capacity and metastatic competency [15]. Phosphorylation of ERK induces expression of cyclin D1 and then promotes activation of E2F transcription factor 1 which appears to be essential in G0/G1-S stage transition [27]. Similarly, OCT1 was discovered to be directly regulated by STAT3 to reduce the expression of caspase 9, BAX, and BAD via ERK and AKT activation to boost proliferation and inhibit apoptosis in esophageal cancer cells [28]. Additionally, OCT1 exhibits to be a regulator of epithelial–mesenchymal transition of malignances [29, 30] and cancer stem cell’s renewal feature [13]. Judging by the observations aforementioned, targeting on OCT1 might be potentially viable for anti-cancer therapy.

Tumor environment, which is a new hotspot in cancer research realms and characterized by hypoxia, nutrition deficiency, low pH, angiogenesis, and so forth [31], is believed to occupy vital position in proliferation, invasion, and metastasis [32]. Khairul et al. [33] uncovered that under insufficient glucose metabolic stress, OCT1, activated by AMPK phosphorylation, negatively regulated miR451 to form a reciprocal feedback loop to assist glioblastoma multiforme cells to survive nutrient/energy starvation. This enlightens us to investigate on OCT1’s effects in CRC microenvironment in our future study. Nonetheless, it should be noted that there are some limitations of our study. First, the finite quantity of subjects with comparatively limited follow-up period is not powerful enough to clarify the role of OCT1 in CRC. Second, the effects of over-expressing OCT1 were not investigated in the present study. Thus, further studies are required to confirm our hypothesis that OCT1 is of favorable prognostic value and a potential target for CRC therapy.

To conclude, this study focuses on the implication of OCT1 in CRC. OCT1 interference inhibits cell reproduction that indicates that the trans-factor participates in colon tumorigenesis and progression. Moreover, abnormally recurrent amplification of OCT1 in CRC and analysis of its level and clinicopathological parameters suggest it as a promising independent predictor of clinical outcome of folks with bowel malignancy.
